# Does correlation heuristic dependence reduce due to classroom teaching? A case study from India

**DOI:** 10.3389/fpsyg.2023.1040538

**Published:** 2023-03-22

**Authors:** Gitanshu Choudhary, Akash K. Rao, Varun Dutt

**Affiliations:** Applied Cognitive Science Lab, Indian Institute of Technology Mandi, Kamand, Himachal Pradesh, India

**Keywords:** stock-and-flow training, correlation heuristic, classroom teaching, stock-flow misperceptations, stock-flow failure, decision making

## Abstract

**Introduction:**

People worldwide have problems understanding the basic stock-flow principles (e.g., correlation heuristic), which govern many everyday tasks. Perhaps, teaching system dynamic concepts in classroom settings might reduce people’s dependence on the correlation heuristic. However, limited literature exists on the effectiveness of classroom curricula in reducing reliance on the correlation heuristic. The present research aims to bridge this gap and empirically understand the effects of classroom teaching programs on reducing people’s reliance on correlation heuristic and improving people’s ability to understand stock-flow concepts. By taking a case from a reputed technology Institute in India, the present research examines how classroom teaching of system dynamics concepts might help students reduce their dependence on the correlation heuristic.

**Methods:**

The experiment consisted of two between-subjects conditions: the experimental and the control (N = 45 in each condition). The experimental condition consisted of randomly registered students that were taught system dynamics principles over 5-months of classroom training. Though, no teaching took place in the control condition. Participants in both conditions were evaluated on their ability to solve stock-flow problems.

**Results:**

Participants in the experimental condition were found to perform better in solving stock-flow problems than subjects in the control condition, and they also relied less on the correlation heuristic.

**Discussion:**

We emphasize the relevance of system dynamics education in graduate curricula in alleviating reliance on the correlation heuristic.

## Introduction

Applying stock-flow knowledge is essential to various real-life choices (Cronin et al., 2009). For example, people make decisions about their caloric consumption (inflow) and their exercise plan (outflow), intending to maintain a healthy weight (stock). Expenditure decisions (outflow) may be made each month based on our earnings (inflow) to maintain an adequate bank balance (stock). In both the cases mentioned above, we try to maintain an adequate stock (for example, healthy weight or adequate bank balance) while considering the flow rates (caloric consumption-exercise or expenditure-earning). The flow rates determine whether a stock gets depleted, accumulated, or remains constant (Sweeney and Sterman, 2000). Relatively common actions like filling a bathtub are governed by stock-and-flow concepts. When the water flow from the source exceeds the water flow from drainage, there is an increase in the bathtub’s water level; when the water outflow exceeds the inflow, the water level falls; and when the water flow equals the outflow, the water level remains constant (Sweeney and Sterman, 2000). Although principles of calculus govern how flows affect stocks, knowing those principles is not essential for understanding the existing stock-flow relationship (Cronin et al., 2009). Research indicates that the population has issues understanding and solving the pervasive nature of stock-and-flow problems in everyday life. Even the highly educated strata of the people with strong managerial and mathematics backgrounds find stock-flow problems difficult and unintuitive (Sterman and Sweeney, 2002; Kumar and Dutt, 2018a).

The inability to comprehend stock-flow problems’ governing principles results in stock-flow failure, i.e., the difficulty in answering stock-flow problems (Cronin et al., 2009). The stock-flow failure is persistent, resulting in wrong problem-solving techniques or heuristics (Moxnes, 2000; Cronin et al., 2009). For example, the correlation heuristic leads individuals to incorrectly assume that a system’s inflow or net flow rate is positively associated with its stock or accumulation. More specifically, individuals disregard the impact of outflows and mistakenly believe that if the system’s inflow rate increases, so will its stock, and vice versa (Sweeney and Sterman, 2000; Dutt and Gonzalez, 2012a, b). Various stock-and-flow problems exhibit the correlation heuristic’s use as a guiding principle (Gonzalez and Wong, 2012; Kumar and Dutt, 2018b). Even though the underlying mathematics for all stock-and-flow problems is the same (Cronin et al., 2009; Rooney-Varga et al., 2020; Béchard et al., 2022; Shin et al., 2022), i.e., the stock is accumulated due to the inflow rate and is depleted due to the outflow rate, the consequences for different stock-flow failures can still vary vastly.

Not all stock-and-flow problems are as trivial as filling a bathtub and managing a bank account. Stock-flow principles also govern large sector industries and the Earth’s climatic system (Kumar and Dutt, 2018a). For example, the accumulation of greenhouse gases (GHG) increases with GHG emissions. It only decreases when the absorption rate exceeds GHG’s emission rate (Dutt and Gonzalez, 2012b). However, because of the correlation heuristic, people might wrongly infer that reducing GHG emissions will decrease GHG concentration in the environment (Dutt and Gonzalez, 2012b).

Moreover, while instantaneous feedback is generally available for simple stock-flow problems like filling a bathtub, i.e., noticing that the bathtub is overflowing, similar immediate feedback is not available for problems like the Earth’s climatic system. Instead, the effect of the rise of GHG in the environment can only be observed in the far future in the form of climatic disasters (Sterman and Sweeney, 2002, 2007). Thus, dependence on faulty problem-solving techniques like the correlation heuristic for crucial systems such as Earth’s climatic system might lead to a wait-and-see behavior towards climate change, further worsening Earth’s atmospheric condition (Kumar and Dutt, 2018a). Due to the significance of stock-and-flow principles in governing essential issues such as climate change, it is necessary to educate and increase people’s conceptual understanding and knowledge of stock-and-flow problems and improve their decision-making abilities (Richardson, 2011; Béchard et al., 2022).

Literature in stock-flow decision making highlights the importance of developing education techniques and efficacious approaches to increase understanding of stock-flow problems (Gonzalez and Wong, 2012; Dutt and Gonzalez, 2012a, 2012b). However, classroom teaching that propagates linear thinking usually is seldom successful in solving problems (Rasmussen, 1983, 1986). For example, prior literature has argued that the conventional education forms could reinforce stock-flow misperceptions instead of alleviating them. Research has found that traditional forms of education fail to deliver and teach the basic concepts of stock and accumulation (Cronin et al., 2009; Sterman, 2010). Furthermore, it might even facilitate the use of the correlation heuristic by promoting the use of linear thinking among students (Ben-Zeev and Star, 2001; Cronin et al., 2009). However, according to Rasmussen’s skills, rules, and knowledge taxonomy (Rasmussen, 1983), a significantly effective way of learning is through acquisition of relevant skills required to execute a corresponding task successfully. Therefore, there is a need to change the pedagogy of stock-flow principles and introduce acquisition of basic stock-flow principles in the education system (Sterman and Sweeney, 2002, 2007).

According to Jenkins et al. (2011), more experiential and real-life-based training is required to effectively develop the ability to solve problems at a fundamental level. Experience and training help people get more acquainted with the systems and concepts. It facilitates problem-solving by helping to grasp better the causal connections between related variables (like stocks and flows) (Dreyfus, 1997; Richardson, 2011). Likewise, the Instance-based Learning Theory (IBLT) suggests that development in our ability to make decisions and solve problems is observed based on our prior experience with similar situations (Gonzalez et al., 2003; Sharma et al., 2018). Furthermore, dynamic thinking also effectively provides insights into solving stock-flow problems (Richmond, 1993; Dorani et al., 2015). Dynamic thinking argues that instead of focusing and predicting the outcome of an event, we should see the event as an ongoing circular process and focus on the behavior patterns of the variables in question. Doing so enables people to frame an issue in behavior over a period of time and better understand the cause-and-effect relationship between variables (Dorani et al., 2015). Thus, by accounting for the behavior of variables over time and understanding their effect on each other, one can better deduce the relationship among associated variables (like stocks and flows) and predict the outcome of a problem (Richmond, 1993).

Based on the theories mentioned above, researchers have developed decision aids to improve people’s understanding of stock-flow systems (refer to Dutt and Gonzalez, 2012a; Kumar and Dutt, 2018a, b). Decision-aids such as Dynamic Climate Change Simulator (DCCS) improve people’s understanding of the stock-flow problem by providing experience in a simulated environment. DCCS and other similar decision aids (refer to Sterman et al., 2012; Chaturvedi et al., 2018) are based on learning transfer. Transfer of learning states that experience and learning influence current and future-leaning and affect the approach to similar or novel problems in the future (Haskell, 2000). Research indicates that decision aids have successfully alleviated people’s biases and misperceptions about stock-flow problems (Dutt and Gonzalez, 2012a; Kumar and Dutt, 2018b).

Similarly, in recent research, Butler et al. (2017) demonstrated that repeatedly retrieving and applying the information to numerous instances is a potent approach for learning that will transfer to various new environments. Therefore, transfer of learning can be a successful approach to improving people’s stock-flow failure. However, fewer steps have been taken to improve the fundamental understanding of stock-flow functions. DCCS, though successful in alleviating misperceptions about the Earth’s climatic system, may not work to improve the fundamental understanding of the stock-flow relationship. Thus, inappropriate heuristics may persist in other stock-flow problems (Dorner, 1997). Overall, there is a need to develop an educational curriculum that aims to improve the fundamental understanding, that results in a holistic improvement of stock-flow relationships and reduces the reliance on inappropriate heuristics and problem-solving methodologies.

Furthermore, recent research conducted in China has shown that students who are well trained in mathematics show fewer stock-flow failures than their western counterparts (Qi and Gonzalez, 2015, 2019). Prior research in stock-flow failure have majorly been carried out in western universities (Sweeney and Sterman, 2000; Sterman and Sweeney, 2002, 2007; Cronin et al., 2009), and research on the stock-and-flow relationship in the developing nations, in particular, in Indian educational institutions is still largely missing in the literature. Therefore, there is also a need to include educational institutes from the developing world and study their curriculum’s effects on people’s stock-flow failures.

Driven by the above observations, the motivation for the present manuscript is to understand the effect of classroom teaching of system dynamics concepts on people’s understanding of stock-flow principles. The classroom teaching is aimed to improve participant’s holistic knowledge of stock-flow principles and not specific context-related knowledge (Kumar and Dutt, 2018a). Also, we conduct this study in the Indian demographic, where such a study has been missing from the system dynamics literature. Most of the prior studies evaluating stock-flow principles have been conducted in the Western countries, especially the United States of America (Sweeney and Sterman, 2000; Sterman and Sweeney, 2002, 2007; Cronin et al., 2009; Sterman, 2010; Harvey et al., 2019; Tadesse and Davidsen, 2019; Rahmandad et al., 2021).

Further, this research aims to examine if the teaching of the stock-flow concepts can be an effective way to reduce reliance on correlation heuristic. Thus, the current research examines how a graduate-level system dynamics course affects students’ comprehension of stock-flow connections (the course details are discussed ahead in this paper). More specifically, we investigate whether participants’ learning from the course improves their problem-solving and fundamental abilities and, as a result, decreases their dependence on the correlation heuristic. To evaluate participants’ understanding of stock-flow relations and their reliance on the correlation heuristic, we relied on tasks proposed by Cronin et al. (2009).

The proposed research contributes to improving our understanding of the effects of classroom teaching of sock-flow concepts on people’s ability to comprehend stock-flow principles. We speculate that the training in relevant system dynamics topics obtained will help participants overcome their reliance on inappropriate heuristics and problem-solving methods used during stock-flow problems. Favorable results may likely support the relevancy of the proposed curriculum in alleviating misperceptions associated with stock-flow failures. Thus, empirical results will contribute to developing a successful pedagogy for stock-flow problems.

In the following part, we summarize the prior work relevant to formulating our hypothesis about how training and experience can help overcome dependence on the correlation heuristic and improve understanding of stock-flow problems. Subsequently, we highlight the design of the present experiment, developed to study the proposed curriculum’s effectiveness in improving participants’ stock-flow failures. Finally, we present our findings and discuss the implications of our outcomes for stock-flow education.

## Background

Prior literature concerning stock-flow failure has established that people have difficulty understanding the principles governing stock-and-flow relationships (Dorner, 1997; Moxnes, 2000). Still, the literature concerning training and the consequent reduction of stock-flow failure is relatively scarce. The conventional form of class education fails to provide the skill set required to answer stock-flow questions (Cronin et al., 2009), and as suggested by Sterman (2010), a comprehensive understanding of stock-flow principles is vital to elucidate the formation of economic plans and determination of market prices. This understanding is especially crucial in economies where one or more commodities are traded simultaneously. In addition, stock-flow principles are essential in understanding health policies in complex economies (like India) with many actors, institutions, and risk factors involved. A deeper understanding of stock-flow principles among young students would help them to design and develop better programs and policies that are aware of and prepared for unexpected situations often encountered in a diverse economy (Bishai et al., 2014). Thus, dedicated programs and educational techniques might be helpful in increasing peoples understanding of stock-flow principles.

In the present times, multiple modes of online (non-conventional) and offline (conventional) teaching have been created (Kanetaki et al., 2021). Several studies have been conducted in the past for evaluating the effectiveness of these conventional and non-conventional methods in facilitating student engagement/arousal and increasing their propensity to accumulate knowledge (Blaine, 2019; Hassan et al., 2020; Moorhouse et al., 2021). For example, e-learning and mobile platforms have gained a lot of traction on the onset of the COVID-19 pandemic (Kanetaki et al., 2021; Coulianos et al., 2022). Krouska et al. (2022) investigated learners’ intention to use mobile phone game-based learning as an alternative educational practice during the COVID-19 pandemic by evaluating pedagogical affordance and student interactions. Results indicated that mobile phone game-based learning had a significant and positive impact on student engagement and academic performance. Troussas et al. (2013) designed “Comulang,” an e-learning system developed to enhance the educational process in computer-based tutoring systems by incorporating collaboration between students and work in groups. Students found the underlying architecture and the interactive nature very interesting and suggested it to be a potentially promising tool towards the creation of distance learning courses. Similarly, Troussas et al. (2021) proposed the “Social Networking for Advancing Knowledge in E-learning environment (SNAKE)” system, which was built as an e-learning software incorporating social characteristics for teaching basic computer programming. Results revealed that participants found the SNAKE system to be more interactive than traditional teaching, enabling them to enjoy the lecture better, even though no significant difference was found in academic performance. Even though e-learning platforms have gained significant popularity over the past couple of years, researchers have highlighted various issues with the same, like less interactive lecturers, frequent change of schedules (which is less frequent in conventional training frameworks), limited internet access, constraints on the practicum course etc. among others (Firmansyah and Triastie, 2020). Even after assuming the logistical issues to be resolved with e-learning, students prefer conventional classroom teaching for subjects with medium-to-high degree of mathematical rigor, or with significant elements of practicum based real-world problem solving, especially in Mathematics, Physics, and Economics (Reyes et al., 2020). Henceforth, classroom teaching is still likely to be relevant and is likely to play a crucial role in imparting science, technology, engineering, and mathematics (STEM) education to students (Wei and Chou, 2020). Thus, offline mode of education still posits required effectiveness to counter stock-flow failures and impart education and training to increase understanding of stock-flow principles.

The pre-existing literature in stock-flow education highlights that learning from experience and training effectively counters stock-flow failures (refer to Dutt and Gonzalez, 2012a; Kumar and Dutt, 2018b). For example, IBLT supports the effectiveness of prior experience and training in facilitating learning about the stock-flow relationship (Gonzalez et al., 2003). As per IBLT, a shift is observed from heuristic-based to instance-based decision making based on the outcomes of our prior experiences with similar situations (Gonzalez et al., 2003). In the stock-flow failure context, learning about the stock-flow relationship’s governing principles through experience and training using models similar to real-life examples is likely to improve one’s future performance in stock-flow problems.

Furthermore, research indicates that the enhanced understanding of the stock-flow principles due to experience and training can be transferred to solving stock-flow problems (Haskell, 2000; Kumar and Dutt, 2018b). For example, Kumar and Dutt (2018b) studied Indian students and used DCCS to provide experience for different structural and surface features of stock-flow problems. Following this, participants were subjected to a stock-flow stabilization task. It was found that the participants in the DCCS conditions performed better in the stock-flow stabilization task than the participants in the no-DCCS conditions. The surface and structural features produced statistically the same results in the stock-flow stabilization task. Thus, the participant’s performance improved due to prior experience in the DCCS simulator tool.

Also, learning about dynamic models or stock-flow failure’s essential features can improve one’s performance in stock-flow problems. For example, a recent study conducted by Fischer and Gonzalez (2016) found that local processing of stock-flow problems showed statistically more reliance on inappropriate problem-solving methods than global processing of stock-flow problems. These results suggested local processing as a reason for stock-flow failure. Learning about such faulty processing systems could improve understanding of correct problem-solving techniques and the ability to avoid faulty processing systems in future instances (Anzai and Simon, 1979; Zhu and Simon, 1988). Based upon the above literature, we hypothesize that:

*H1*: The proportion of correct responses in the stock-flow problem will be more significant in conditions where training is provided than in the absence of training.

Attempts have been made to reduce reliance on faulty heuristics, such as correlation heuristic, while solving stock-flow problems. Prior research has shown that training in principles of system dynamics and experience through a simulated setting can significantly reduce dependance on the correlation heuristic (see Sterman, 2010; Sterman et al., 2012; Dutt and Gonzalez, 2012a). For example, in a study by Sterman (2010), students of a reputed education institute with prior training in mathematics and management were taught system dynamics basics. The study concluded that the training provided in basic principles of system dynamics resulted in a statistically significant reduction in participants’ dependence on the use of the correlation heuristic when trying stock-flow problems (Sterman, 2010). There was also an overall improvement in performance, as the proportion of incorrect responses decreased significantly.

Previously it was believed that prior domain knowledge had little to no effect on performance in the stock-flow task (Brunstein et al., 2010). However, new findings suggest that relevant education and training can improve one’s performance in stock-flow tasks and reduce reliance on the correlation heuristic (Qi and Gonzalez, 2015, 2019). Qi and Gonzalez (2015, 2019) showed that Chinese students with better mathematical knowledge revealed a significantly better understanding of stock-flow tasks. Furthermore, Qi and Gonzalez (2015, 2019) found that Chinese students performed better in stock-flow problems than their counterparts in western education institutes. Overall, the work of Qi and Gonzalez (2015, 2019) highlights the importance of training and curriculum in alleviating stock-flow failures.

Lastly, as already discussed, simulation tools such as Climate Rapid Overview And Decision Support (C-ROADS) and DCCS have also been successfully used to lessen people’s dependence on correlation heuristic during stock-flow tasks (Sterman et al., 2012; Kumar and Dutt, 2018a, b). Such simulation tools have successfully used prior experience and practice sessions in a simulated setting to expand people’s understanding of dynamic models and improve their performance in tasks governed by stock-flow principles. Based on the above-cited literature, we hypothesize that:

*H2*: The proportion of reliance on the correlation heuristic will be less in conditions where training is provided than in the absence of training.

## Methods

### Participants

Overall, 90 participants (29 females and 61 males; mean age = 21.3 years, SD = 1.45 years) at the Indian Institute of Technology (IIT) Mandi, Himachal Pradesh, India, took part in this study. The study was approved by the Institutional Ethical Committee (IEC) at the Indian Institute of Technology Mandi. Participation in the study was voluntary. All the participants gave a written consent form before participating in the study. All the participants were from a Science, Technology, Engineering, or Mathematics (STEM) background. Ninety three percent of participants pursued undergraduate engineering degrees, and 7% pursued graduate master’s and Ph.D. degrees.

### The stock-flow tasks

**Figure 1:**
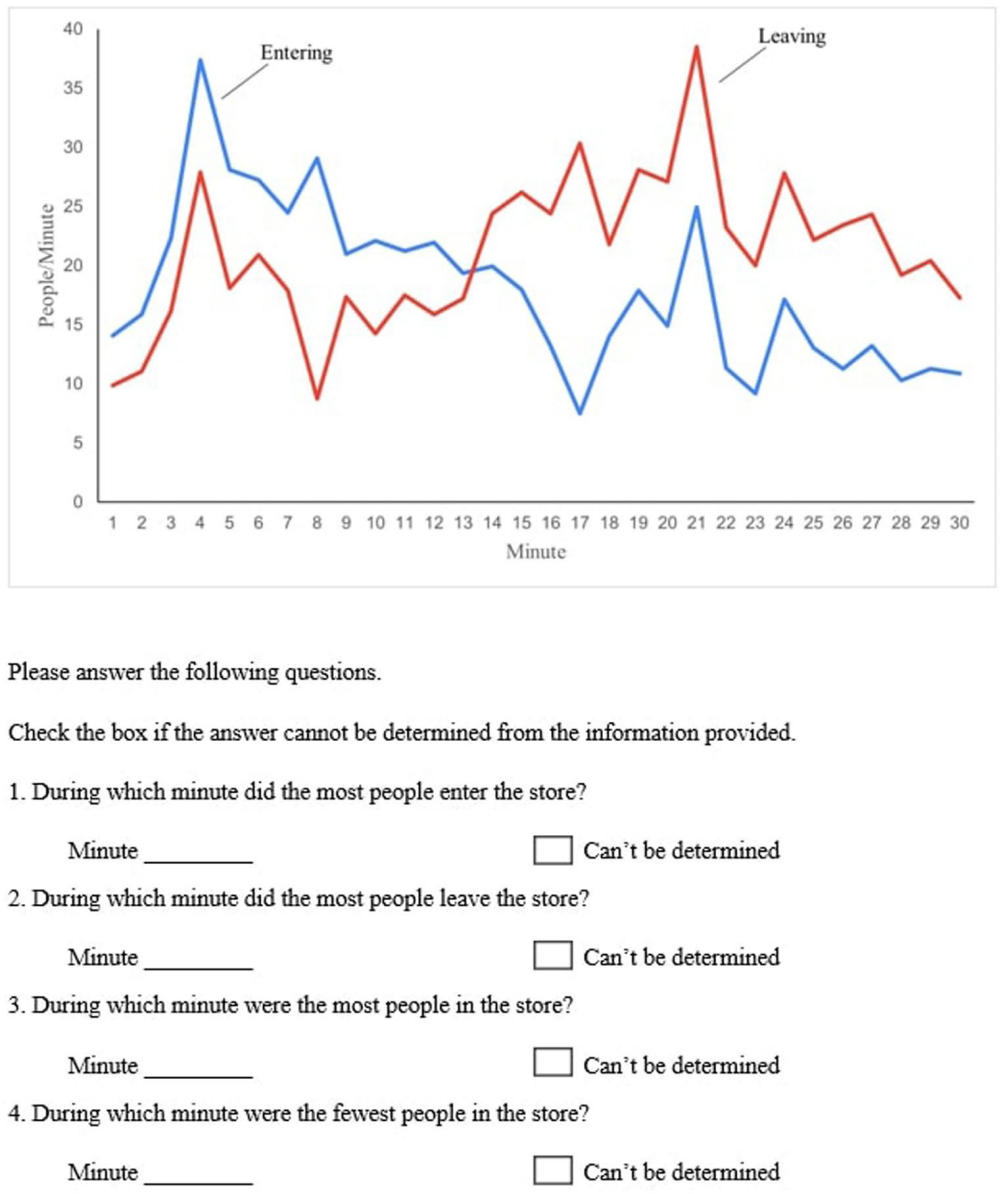
The graph and the corresponding questions are given in task 1.

Participants were presented with two stock-flow tasks. Both the tasks were taken from prior work on stock-flow problems (Cronin et al., 2009). Task 1 was called the “department store” task. As shown in Figure 1, in task 1, participants were given a graph depicting the count of people going in and out of a room every minute over a 30-min period (Sterman and Sweeney, 2002; Cronin et al., 2009). Task 1 consisted of four questions (refer to Figure 1), and based on the graph, participants were asked to answer all four questions. The first two questions tested whether the participants understood the graphs and could differentiate between the inflow and outflow. The last two questions tested whether participants could infer the behavior of the stock over the 30-min interval based on the inflow and outflow graphs.

**Figure 2:**
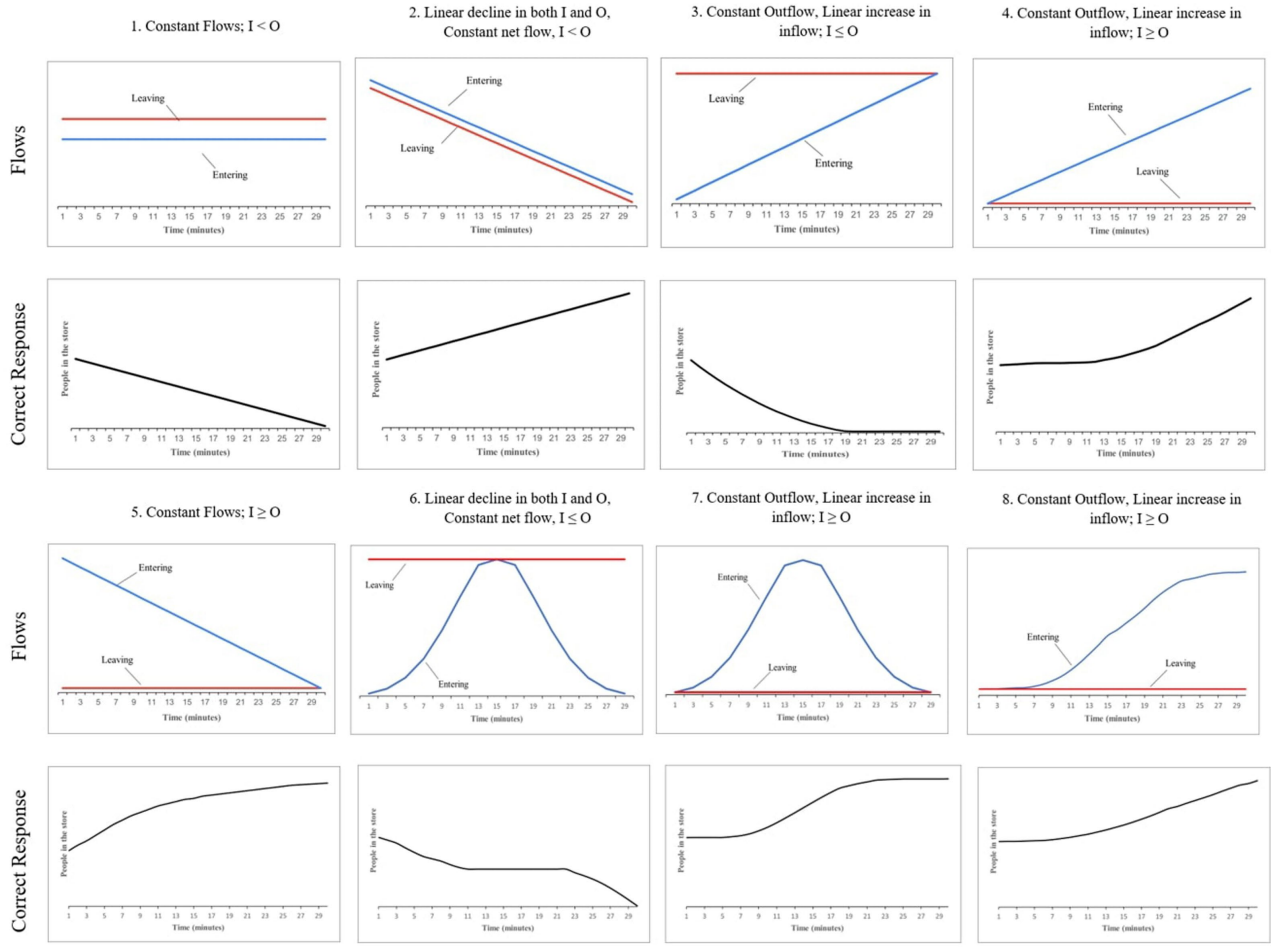
The eight stock-and-flow questions given in task 2 and the corresponding correct responses.

Task 2 was an extension of the department store task presented in task 1. Task 2 consisted of eight questions relating to typical stock-flow problems’ (refer to Figure 2; Cronin et al., 2009). All questions aimed to examine the subject’s comprehension, familiarity with accumulation, and level of correlation heuristic assumption. Similar to task 1, task 2 gave participants a graph showing how many people entered and left a room every minute over a 30-min period (Cronin et al., 2009). However, unlike task 1, in task 2, as per the graph provided, subjects were instructed to draw the number of people present in the store over a 30-min interval on a blank chart placed underneath the flow graph. The sketching of the stock over a 30-min interval helped study whether the participants relied on the usage of the correlation heuristic while trying the stock-flow problems. Participants had to start from the black dot on the y-axis of the blank stock graph and sketch the trajectory of the number of people in the store over time. The point for initiating the graph drawing was placed midpoint on the vertical axis to avoid potential biases.

The eight questions presented to the participants in task 2 were of increasing difficulty, i.e., question 1 (Q1) was relatively more straightforward than Q2, while Q8 was deemed the hardest amongst the eight questions. As shown in Figure 2, in the former questions (Q1 to Q5), the inflow rate was kept constant over a 30-min time interval, or a linear increase or decrease was observed in the inflow rate over a 30-min time interval. A dynamic inflow rate was followed in the latter questions (Q6 to Q8), contributing to the questions’ difficulty. The outflow rate was constant or only linearly changing in all the questions.

### Experimental design

The experiment consisted of two between-subject conditions: experimental and control. A total of 90 students applied to register to a graduate-level course on computational modeling of social systems (CMSS). The CMSS course was offered at IIT Mandi in the spring of 2019. A set of 45-students were randomly selected to register in the CMSS course by the computer (experimental condition) and the remaining students’ registrations were rejected from the course (control condition). The mean age of participants in the experimental condition was 21.8 years, 33 were males and the remaining were females. Of the 45 participants in the experimental condition 41 were enrolled in an undergraduate degree while the remaining students were pursuing their post-graduate degree. The mean age of participants in the control condition was 20.8 years, 28 were males and the remaining were females. Of the 45 participants in the control condition 43 were enrolled in an undergraduate degree while the remaining students were pursuing their post-graduate degree. The experimental condition included 42 h of teaching in the CMSS course. The CMSS course was intended to familiarize students with arithmetical and system-dynamic methods of modeling the actions of simple and complex social systems. Importance was given to those computational models and techniques which enable modelers to capture several social systems’ dynamics at the aggregate level. The whole course was spread over 5 months. The course included a heterogenous mixture of conventional instruction (through PowerPoint® presentations), lab-based instruction on the design and interpretation of system dynamics models through the VensimPLE® software (García, 2020), numerical problem-solving, assignments, and projects. More details on the course can be found in the Appendix. Participants in the control condition were provided with no such exposure or training in the CMSS course.

Data from the experimental and control conditions were acquired at three instances during the semester. The data for task 1 was collected at the start of the semester, i.e., the second week of February 2019. Task 1 helped determine any pre-existing differences between the experimental and control groups regarding knowledge about the stock-flow problems. Task 1 also helped determine if the participants could correctly read the graphs and differentiate between the inflow and the outflow rates. Participants in the experimental and control groups were given 15 min to answer the four questions in task 1.

The data for task 2 was collected in two halves. The data for Q1 to Q4 was gathered during the mid-semester point, i.e., the first week of April 2019. By the mid-term of the semester, the course instructor had finished concepts up to Module 3 (as shown in Appendix). The data for Q5 to Q8 was collected at the endpoint of the semester, i.e., the second week of June 2019. By the semester’s end, the course instructor had finished all six modules (as shown in the Appendix). Task 2 helped in studying the effectiveness of the CMSS course in increasing the participant’s ability to solve stock-flow problems. For each half of task 2 (comprising of four questions), participants were asked to answer the four questions within 30-min. The control condition participants who were not provided any of the CMSS course training were also given the same amount of time for both halves of task 2.

Therefore, the exposure and teachings provided through the CMSS practicum acted as the independent variable, and the dependent variable was the rightness of the participants’ responses. Like Cronin et al. (2009), a very lenient scoring was done. The responses for task 1 had a clear right or wrong answer, while the responses for task 2 were judged on qualitative rather than quantitative characteristics. So, even if a participant drew an answer that was not quantitively correct or began from a point other than the initial point provided in the blank graph, they were not penalized. A response was deemed correct if consistent with the basic stock-and-flow principle. For example, the stock rose when the inflow was more significant than the outflow, decreased when the outflow was greater than the inflow, and was constant when the inflow equaled outflow. Thus, if the rate of change of the stock accumulation was correctly corresponding to the inflow and outflow rate provided in the questions, then such a response was correct.

We requested the assistance of two unbiased judges to evaluate the participant’s responses to task 2. The judges were post-graduate level students with a working knowledge of stock-and-flow concepts and were provided with the correct answer to each of the eight questions. In the initial round of evaluation, the judges were asked to grade each response and submit their responses. After the initial round of evaluation, if there were differences in the judges’ opinions on responses, these differences were to be discussed and resolved among the judges. However, no such discussion was needed after the initial evaluation process as there were no disagreements between the judges’ evaluations.

Responses in the two between-subjects condition were translated to codes for computational purposes. The correct answers were rated 1, while the wrong ones were rated 0. Additionally, for task 2, wrong answers that displayed the correlation heuristic were rated as 1, while incorrect answers that did not show the correlation heuristic were rated as 0. For task 2, we used binary coding in two separate instances. First, we used the aggregate responses to determine the proportion of incorrect and correct answers. Second, we used the coding to determine the proportion of participants showing the correlation heuristic. For computational purposes, the 0 and 1 responses were aggregated.

Overall, we expected the experimental condition to outperform the control condition on account of learning transfer (Perkins and Salomon, 1992; Haskell, 2000) and IBLT (Gonzalez et al., 2003). Also, we expected the experimental condition to be less affected by the correlation heuristic than the control condition.

### Research design

**Figure 3:**
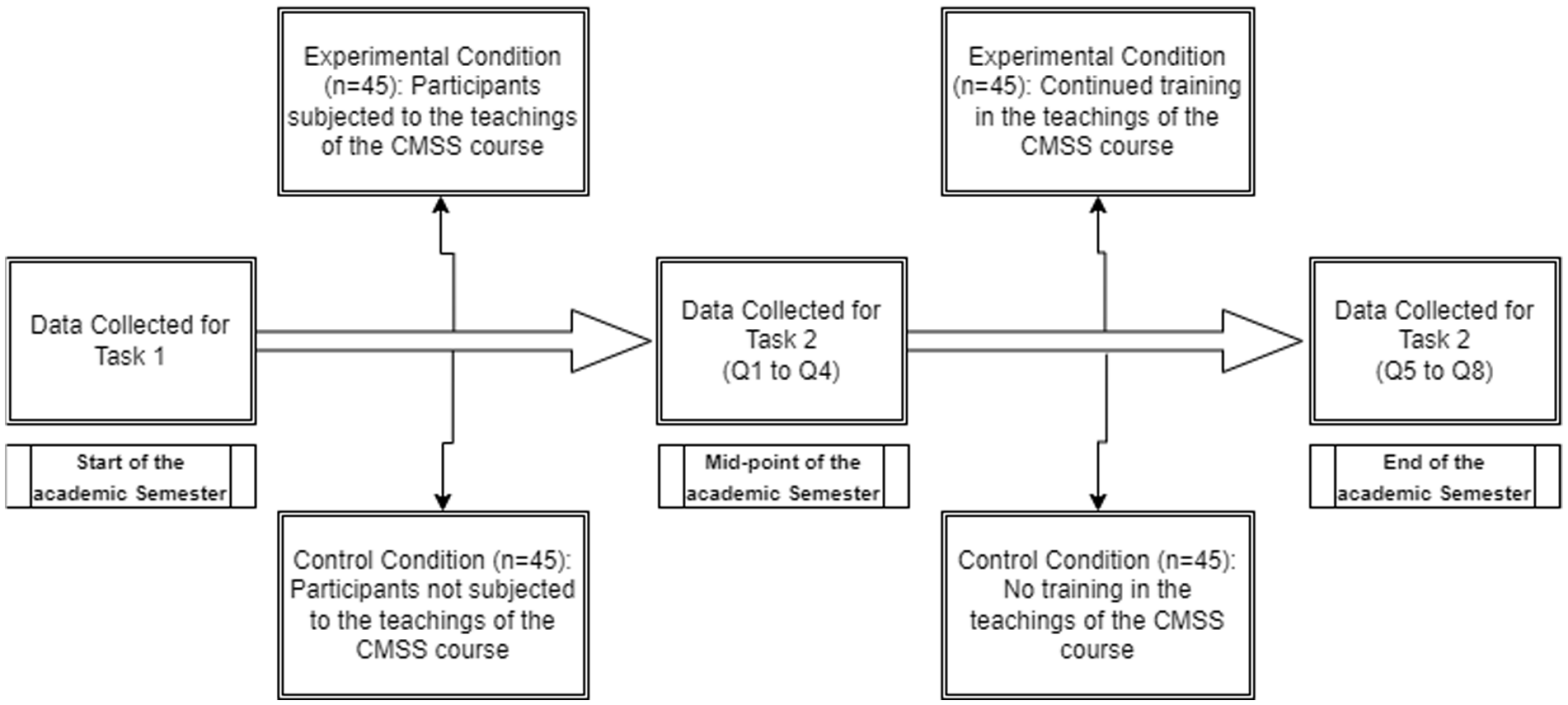
Research schema outlining the experimental design and methodology employed in the study.

A two between-subject condition (experimental and control) were used to study the effectiveness of CMSS course’s teaching in improving participants’ understanding of stock-flow principles and reducing reliance on correlation heuristic. Participants in the experimental condition were provided exposure and training in stock-flow concepts through the CMSS course, while participants in the control condition were provided with no such training. As previously stated, data was collected a total of three times from the experimental and the control group, the data for determining baseline differences between the experimental and the control group was collected at the start of the course. Subsequently, data was collected two more times, first at the mid-point of the course and secondly at the end of the course, to analyze the effectiveness of the CMSS course (refer to Figure 3). Participants’ data in both conditions were acquired under a similar setup of physical factors like time and sitting space. Two unbiased judges were used to evaluate the participant’s responses. Based on the judge’s evaluations, participants’ responses were coded either as correct or incorrect and, if incorrect, whether reliance on correlation heuristic was exhibited or not exhibited (as detailed in the above section).

### Data analyses

We used the chi-square test, a statistical technique used to determine the association between two or more categorical variables, to compare whether the performance across two conditions was significantly different (Field, 2013). The alpha level (the probability of rejecting the null hypothesis when it was true) was set at 0.05 (or 5%). Thus, the null hypothesis was rejected if the value of p (indicating the evidence in favor of the null hypothesis when true) was less than the alpha level (0.05). We performed two experimental conditions × two response outcomes chi-square tests to study whether the incorrect outcomes across the experimental and control conditions were independent or related. Furthermore, for task 2, we performed two chi-square tests on the experimental conditions against the two correlation heuristic outcomes to evaluate the proportion of correlation heuristic following in the incorrect answers across the experiment and control conditions.

## Results

### Task 1

Table 1 shows the results for each of the four questions presented in task 1. For Q1, all the experimental and control groups participants answered the question correctly. Therefore, no chi-square statistics were computed for Q1 due to the identical responses in the experimental and control conditions. For the remaining three questions, i.e., Q2, Q3, and Q4, no significant difference was observed in the proportion of correct responses in the experimental and control conditions. These findings indicate that there was no significant difference in stock-flow problem-solving skills of the participants of the experimental group and control group before the start of the CMSS course.

**Table 1:** Results involving a proportion of correct responses across different questions and conditions in task 1.

Question	Condition comparisons	*χ*^2^(1)	*p*
1	CR-exp (1.00) ∼ CR-con (1.00)	*	*
2	CR-exp (0.98) ~ CR-con (0.93)	1.047	0.31
3	CR-exp (0.18) ~ CR-con (0.20)	0.073	0.79
4	CR-exp (0.18) ~ CR-con (0.16)	0.080	0.78

CR-exp and CR-con represent “correct response in experimental conditions” and “correct response in control conditions,” respectively. The number in the bracket represents the proportion of participants in each condition. The symbol ∼ indicates that the proportions in the two conditions were similar. The symbol * indicates that the proportions in the two conditions were identical; therefore, no statistics were computed.

### Task 2

Table 2 shows the results for the eight questions presented in task 2 and the combined effects. For six out of eight questions, i.e., Q2, Q3, Q5, Q6, Q7, and Q8, a significantly higher proportion of correct answers were recorded in the experimental than in the control condition. These findings are as per our expectations. For the remaining two questions, i.e., Q1 and Q4, no significant difference was observed in the proportion of correct responses in the experimental and control conditions. These findings are not as per our expectations.

**Table 2:** Results involving proportion of correct responses and reliance on correlation heuristic among participants across different questions and conditions in task 2.

Question	Condition comparisons	*χ*^2^(1)	*p*
1	CR-exp (0.69) ∼ CR-con (0.58)	1.196	0.27
	CH-exp (0.36) < CH-con (0.90)	10.483	< 0.01
2	CR-exp (0.64) > CR-con (0.44)	3.629	< 0.05
	CH-exp (0.63) ∼ CH-con (0.80)	1.522	0.22
3	CR-exp (0.38) > CR-con (0.11)	8.663	< 0.01
	CH-exp (0.43) < CH-con (0.78)	8.502	<0.01
4	CR-exp (0.22) ~ CR-con (0.09)	3.045	0.08
	CH-exp (0.51) < CH-con (0.85)	10.304	<0.01
5	CR-exp (0.98) > CR-con (0.38)	37.089	<0.01
	CH-exp (0.00) < CH-con (0.93)	8.976	<0.01
6	CR-exp (0.82) > CR-con (0.27)	27.999	< 0.01
	CH-exp (0.75) ~ CH-con (0.91)	1.522	0.22
7	CR-exp (0.76) > CR-con (0.16)	32.658	< 0.01
	CH-exp (0.91) ~ CH - con (0.90)	0.019	0.89
8	CR-exp (0.62) > CR-con (0.13)	22.878	< 0.01
	CH-exp (0.94) ~ CH-con (0.87)	0.596	0.44
Q1 to Q8	CR-exp (0.64) > CR-con (0.27)	99.105	< 0.01
	CH-exp (0.59) < CH-con (0.86)	36.422	<0.01

CR - exp and CR - con represent “correct response in experimental conditions” and “correct response in control conditions,” respectively. CH-exp and CH-con represent “correlation heuristic in the incorrect responses of experimental conditions” and “correlation heuristic in the incorrect responses of control conditions,” respectively. The number in the bracket represents the proportion of participants in each condition. The symbol ∼ indicates that the proportions in the two conditions were similar to each other.

Furthermore, for four out of eight questions, i.e., Q1, Q3, Q4, and Q5, a significantly lower proportion of incorrect responses exhibited the use of the correlation heuristic in the experimental than in the control condition. These findings are as per our expectations. For the remaining four questions, i.e., Q2, Q6, Q7, and Q8, no significant difference was observed for reliance on the correlation heuristic in the incorrect responses of the experimental and control conditions. These findings are not as per our expectations.

Overall, for all eight questions put together, the proportion of correct answers in the experimental condition was significantly higher than in the control condition. This result meets our expectations. Additionally, the proportion of wrong answers that exhibited the use of the correlation heuristic for the problem was significantly lower in the experimental condition than in the control condition. This result meets our expectations.

**Figure 4:**
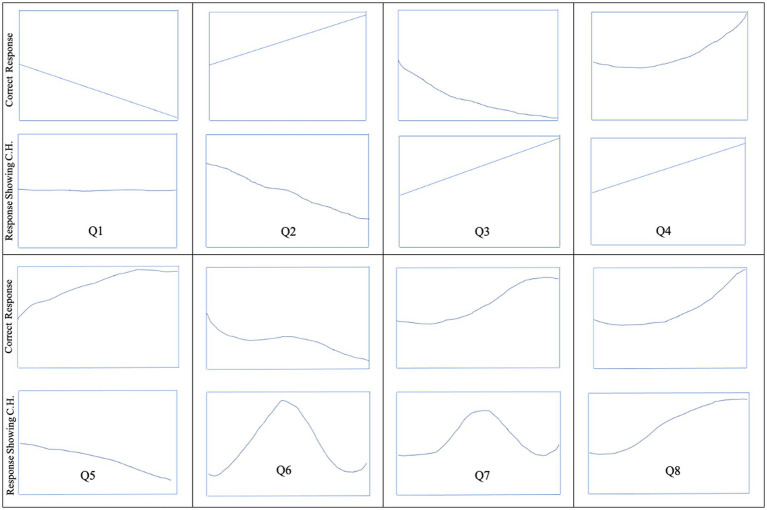
Participant’s typical sketch for correct response and response showing correlation heuristic to the eight questions.

Figure 4 shows the typical response of participants for each of the eight questions in task 2. The obtained responses, in general, are as per our expectations.

## Discussion

The present study’s objective was to examine the effect of exposure and training in a system dynamics course on participants’ understanding of the stock-and-flow behavior. Two tasks motivated from prior literature, i.e., task 1 and task 2, consisting of four and eight problems, were used for assessment purposes. Task 1 determined any pre-existing differences between the experimental and control groups regarding graph reading ability and stock-flow problems. No significant difference was observed between the experimental and control groups for any of the four questions of task 1. Thus, indicating a homogeneity between the experimental and control groups regarding knowledge about the stock-flow problems before the CMSS course.

We hypothesized that the exposure and training in the CMSS course conducted in a formal setting would increase participants’ understanding of the stock-flow relationship and reduce their reliance on the correlation heuristic. Task 2 was used to assess the effectiveness of the CMSS course in increasing the participant’s ability to solve the stock-flow problems. The obtained results provided support to our assumptions.

As per our expectation for Q2, Q3, Q5, Q6, Q7, and Q8 of task 2, the proportion of correct answers for the experimental condition was significantly higher compared to the control condition. These results support the effectiveness of learning transfer (Haskell, 2000) and IBLT (Gonzalez et al., 2003) in improving future performances. The CMSS course teachings may have made participants aware of the governing principles of system dynamics and helped them understand how a system’s inflow and outflow rate affect its stock. Moreover, the teaching may have helped participants avoid the counterintuitive nature of the questions, which prompted wrong problem-solving techniques. In contrast, participants with no prior training in stock-flow relations may have used the wrong problem-solving techniques (Cronin et al., 2009). Thus, this resulted in a significantly greater proportion of correct answers in the experimental condition compared to the control condition.

In contrast to our expectation for Q1 and Q4 of task 2, the proportion of correct responses in experimental and control conditions was not significantly different. One likely reason for the insignificant differences can be the question’s difficulty level. Difficult questions generally produce more cognitive load, resulting in faulty problem-solving techniques and poor performance (Sweller, 1994). Q1 and Q4 were relatively easy questions present in our study, requiring less understanding of stock-flow principles. Thus, they may not have resulted in a cognitive load sufficient to elicit statistically poor performance in the control condition (Sweller, 1994; De Jong, 2010).

Moreover, based on the recent research by Qi and Gonzalez (2015, 2019), one expects a statistically better performance of students in stock-flow problems due to better knowledge of mathematical concepts. We can argue that all the participants in the present study’s control condition belonged to the STEM background and were enrolled in one of India’s top educational institutes. Thus, due to past education, we could expect good performance from such students in the stock-flow problems (Qi and Gonzalez, 2015, 2019), especially when the problems were relatively easy. Overall, prior domain knowledge coupled with lower cognitive load could explain the statistically similar performance in the control and experimental conditions for Q1 and Q2.

Per our expectation, for Q1, Q3, Q4, and Q5 of task 2, there was a significant reduction in reliance on the correlation heuristic in the experimental condition compared to the control condition. These results support prior research on the effectiveness of prior exposure and training in system-dynamics principles in reducing stock-flow failures and the usage of the correlation heuristic (Sterman, 2010). The knowledge and training provided to the students enrolled in the CMSS course may have improved their understanding of the stock-flow relations. Moreover, participants must have been aware of the faulty perceptions and biases in stock-flow problems. As a result, trained participants may have avoided using the correlation heuristic to solve the stock-flow problem. Therefore, the enhanced knowledge of stock-flow relations may have translated into reduced reliance on the correlation heuristic through the transference of learning (Butler et al., 2017).

In contrast to our expectation for Q2, Q6, Q7, and Q8, of task 2, there was no significant reduction in reliance on the correlation heuristic in the experimental compared to the control condition. We expect encoding failure to be a likely reason for the insignificant effect of training in reducing reliance on the correlation heuristic. According to IBLT (Gonzalez et al., 2003), there is a shift from heuristic-based thinking to instance-based thinking based on previous interactions with similar situations. However, if we cannot recall the last similar instances or their outcomes, such experiences will not affect our future decisions. So, if a student failed to pay attention during the CMSS course, they will not be able to recall the concepts necessary for solving the problem (Haskell, 2000; Gonzalez et al., 2003). As a result, they will be forced to use inappropriate problem-solving techniques, such as the correlation heuristic, to answer the cognitively demanding stock-flow problems.

Moreover, for the relatively harder questions (Q6-Q8), participants in both experimental and control conditions majorly relied on the correlation heuristic when responding to the questions incorrectly. Therefore, resulting in an identical proportion of participants in both the conditions using the correlation heuristic while answering the questions incorrectly. Thus, lack of attention and the difficulty level of questions may have led to a statistically similar proportion of the correlation heuristic reliance on the control and experimental condition’s wrong responses.

Furthermore, for task 2, there was a significant consequence of prior exposure and training in increasing the overall proportion of correct responses and reducing the reliance on the correlation heuristic for problem-solving. The combined analysis of all eight questions revealed that the participants in the experimental condition performed significantly better and exhibited less reliance on correlation heuristic than the participants in the control condition. Based on the findings, the teaching delivered in the CMSS course may have helped students better comprehend the stock-flow principles. The use of the correlation heuristic may have greatly decreased, and problem-solving abilities may have improved due to a better knowledge of the stock-flow tenets.

The results support previous classroom-based research, highlighting the effectiveness of classroom training in reducing stock-flow failure (Cronin et al., 2009; Sterman, 2010). Like Sterman (2010), the present study also assessed the effect of the CMSS course, an introductory system dynamics course, on participants’ ability to understand stock-flow principles and reduce reliance on correlation heuristic. Similar to the findings presented by Sterman (2010), the current study also exhibited a positive and significant effect of classroom teaching of stock-flow principles in improving overall performance in stock-flow questions and reducing reliance on correlation heuristic (Cronin et al., 2009; Sterman, 2010). Though the percentage of overall incorrect responses noted in Sterman (2010) work was relatively less than those reported in this study. A primary cause for relatively fewer incorrect responses could be attributed to the fact that subjects enrolled in the system dynamics course offered by Sterman (2010) were students from the field of management, which is a related field to system dynamics. However, participants in this study were all from STEM backgrounds. Thus, the participant’s prior education background may have facilitated their ability to understand stock-flow principles better during the system dynamics course (Cronin et al., 2009; Sterman, 2010). Furthermore, following Qi and Gonzalez (2015, 2019), we also studied a different demographic of the world to understand the effect of demographic and prior education on people’s inability to solve stock-flow problems. Lastly, our results also agree with simulation tool’s literature, where educational tools like the dynamic climate change simulator (DCCS) have been used to communicate stock-flow principles among people (Kumar and Dutt, 2018a).

## Conclusion

Overall, the results support training effectiveness in the system-dynamics course in alleviating misperceptions concerning stock-flow behavior. As previously stated, the results align with the successful results of DCCS training in improving participants’ performance in the stock-flow stabilization task in a different study (Kumar and Dutt, 2018b). The current experiment results highlight that training and experience in the relevant domain area can improve one’s reliance on inappropriate problem-solving methods, such as the correlation heuristic (Haskell, 2000). Therefore, significant improvements in performance facilitated by formal training in academic settings agree with some studies supporting education’s role in reducing stock-flow failures (see Sterman, 2010; Qi and Gonzalez, 2015, 2019). Furthermore, the present study contributes to the research on stock-flow failure. Most studies on stock-flow failure have been carried out in western countries (Sterman, 2010; Rooney-Varga et al., 2020; Rahmandad et al., 2021; Béchard et al., 2022). In relative terms, there is hardly any literature on stock-flow failures present in the educational institutes of the developing world (e.g., India). To the best of our knowledge, this is the first study to be conducted among the Indian STEM student demographic. India has a significantly large and ethnically more diverse population compared to most other countries in the world (Duppati et al., 2020), with the students hailing from different cultural/financial/regional backgrounds. The authors believe that the current study in the Indian context increases the generalizability of the findings. Lastly, the course material used in teaching the basics of system dynamics to enrolled students can build a pedagogy of stock-flow and system dynamics principles. This effective form of training can go a long way in improving people’s understanding of many essential systems governed by stock-flow principles, such as Earth’s climatic system.

To summarize, the present manuscript firstly consolidates and supports the effectiveness of classroom training in reducing reliance on faulty problem-solving techniques. It introduces the Indian demographic to the literature, which was previously missing. Finally, it also provides a new and effective pedagogy that can help educate people and increase their understanding of stock-flow principles.

One limitation of the present study is that the participant’s prior academic background and interest in the teachings of the CMSS course may have amplified the curriculum’s effectiveness for system-dynamics training (Qi and Gonzalez, 2019). In contrast, participants’ prior experience with the conventional form of education may have tampered with the teachings of the CMSS course, resulting in downgrading the effectiveness of the curriculum used (Ben-Zeev and Star, 2001; Cronin et al., 2009). One way to overcome this limitation is to study training effectiveness *via* the system-dynamics course on participants from non-STEM backgrounds *via* a pre-post design. Secondly, in the current manuscript, we do not directly compare the same classroom teaching curriculum’s effectiveness on different demographics worldwide. It will be interesting to study the effects of the same curriculum on peers of different demographics with unique educational and cultural backgrounds.

Moreover, in our future studies, we plan to incorporate more stock-flow problems of varying nature (Kumar and Dutt, 2018b) to study if general training in stock-flow principles can translate to a better understanding of more complex stock-flow systems like Earth’s climatic system. Extended versions of the CMSS course can also be incorporated across multiple academic semesters to incorporate multiple stock-flow questions and cross-check the reliability and validity of the results. Also, we plan to examine if training can have any unwanted effect on an individual’s stock-flow problem-solving skills. The training was ineffective in producing significant results for all the questions. It is possible that the training may have fostered a false sense of knowledge, making participants prone to overconfidence bias and affecting their attempt to solve certain stock-flow problems. Thus, future research should focus on the downsides of training, if any. Finally, we plan to cover a larger population sample, with varied questions to further enhance the results’ generalizability and ensure it is reliability and validity. In conclusion, the current manuscript is a building block toward the bigger goal of developing educational curricula which could reduce inappropriate problem-solving techniques in stock-flow problems.

## Data availability statement

The raw data supporting the conclusions of this article will be made available by the authors, without undue reservation.

## Ethics statement

The studies involving human participants were reviewed and approved by Institutional Ethical Committee (IEC) at the Indian Institute of Technology Mandi. The patients/participants provided their written informed consent to participate in this study.

## Author contributions

GC analyzed the results obtained and complied the manuscript. AR acquired the data for the aforementioned experiment. VD helped in writing the manuscript and guided the experiment. All authors contributed to the article and approved the submitted version.

## Funding

This work was supported by the Indian Institute of Technology Mandi, who provided the necessary financial and computational resources for this experiment.

## Conflict of interest

The authors declare that the research was conducted in the absence of any commercial or financial relationships that could be construed as a potential conflict of interest.

## Publisher’s note

All claims expressed in this article are solely those of the authors and do not necessarily represent those of their affiliated organizations, or those of the publisher, the editors and the reviewers. Any product that may be evaluated in this article, or claim that may be made by its manufacturer, is not guaranteed or endorsed by the publisher.

## Appendix

The link to the syllabus of the Computational Modeling on Social Systems (CMSS) taught at Indian Institute of Technology Mandi can be found here: https://drive.google.com/file/d/1EK347X9fdlb0_JdUFio97AVRch1epQqU/view?usp=sharing

The textbook being referred was “Business Dynamics: Systems Thinking and Modeling for a Complex World” by John D. Sterman, distributed by Irwin-McGraw Hill publishers.

As mentioned in the syllabus, the material for module 1 (Learning in complex systems) was taken from part 1, section 1 (pp. 3–38) of Sterman’s book. The material for module 2 (The modeling process) was taken from part 1, section 3 (pp. 84–103), part 1, section 4 (pp. 107–133), part 2, section 7 (pp. 231–258), and part 2, section 8 (263–290). The material for module 3 (Applications of modeling process) was taken from part 3, section 9 (pp. 293–339). The material for module 4 (Feedbacks and delays) was taken from part 3, section 8 (pp. 263–290). As part of modules 5 and 6, the course instructor explained and executed some system dynamics models in VensimPLE® (a popular simulation software) such as generic bass model, supply and demand model, pendulum model, HIV-AIDS epidemic model among others. In addition, a case study titled “Explaining Human Behavior in Dynamic Tasks through Reinforcement Learning” (ISSN 1798–2,340) by Dutt (2011), which encapsulated the efficacy of different reinforcement learning algorithms in modelling behavior in dynamic stock and flow tasks, was also taken up.

The detailed course materials, including lecture slides, assignments, and quizzes will be made available to other potential future collaborators on request.
